# Human babesiosis: Indication of a molecular mimicry between thrombospondin domains from a novel *Babesia microti* BmP53 protein and host platelets molecules

**DOI:** 10.1371/journal.pone.0185372

**Published:** 2017-10-17

**Authors:** Ahmed Abdelmoniem Mousa, Daniel Barry Roche, Mohamad Alaa Terkawi, Kyohko Kameyama, Ketsarin Kamyingkird, Patrick Vudriko, Akram Salama, Shinuo Cao, Sahar Orabi, Hanem Khalifa, Mohamed Ahmed, Mabrouk Attia, Ahmed Elkirdasy, Yoshifumi Nishikawa, Xuenan Xuan, Emmanuel Cornillot

**Affiliations:** 1 Institut de Biologie Computationnelle (IBC), LIRMM, CNRS, Université de Montpellier, Montpellier, France; 2 National Research Center for Protozoan Diseases, Obihiro University of Agriculture and Veterinary Medicine, Obihiro, Hokkaido, Japan; 3 Department of Biochemistry and Chemistry of Nutrition, Faculty of Veterinary Medicine, University of Sadat City, Sadat City, Menoufia, Egypt; 4 Centre de Recherche en Biologie cellulaire de Montpellier, CNRS-UMR 5237, Montpellier, France; 5 Department of Parasitology, Faculty of Veterinary Medicine, Kasetsart University, Bangkok, Thailand; 6 Department of Animal Medicine and Infectious Diseases, Faculty of Veterinary Medicine, University of Sadat City, Sadat City, Menoufia, Egypt; 7 Institut de Recherche en Cancérologie de Montpellier (IRCM-INSERM U1194), Institut régional du Cancer Montpellier (ICM) and Université de Montpellier, Montpellier, France; Center for Cancer Research, UNITED STATES

## Abstract

Human babesiosis is caused by the apicomplexan parasite *Babesia microti*, which is of major public health concern in the United States and elsewhere, resulting in malaise and fatigue, followed by a fever and hemolytic anemia. In this paper we focus on the characterization of a novel *B*. *microti* thrombospondin domain (TSP1)-containing protein (BmP53) from the new annotation of the *B*. *microti* genome (locus 'BmR1_04g09041'). This novel protein (BmP53) had a single TSP1 and a transmembrane domain, with a short cytoplasmic tail containing a sub-terminal glutamine residue, but no signal peptide and Von Willebrand factor type A domains (VWA), which are found in classical thrombospondin-related adhesive proteins (TRAP). Co-localization assays of BmP53 and *Babesia microti* secreted antigen 1 (BmSA1) suggested that BmP53 might be a non-secretory membranous protein. Molecular mimicry between the TSP1 domain from BmP53 and host platelets molecules was indicated through different measures of sequence homology, phylogenetic analysis, 3D structure and shared epitopes. Indeed, hamster isolated platelets cross-reacted with mouse anti-BmP53-TSP1. Molecular mimicry are used to help parasites to escape immune defenses, resulting in immune evasion or autoimmunity. Furthermore, specific host reactivity was also detected against the TSP1-free part of BmP53 in infected hamster sera. In conclusion, the TSP1 domain mimicry might help in studying the mechanisms of parasite-induced thrombocytopenia, with the TSP1-free truncate of the protein representing a potential safe candidate for future vaccine studies.

## Introduction

*B*. *microti* is a protozoan apicomplexan piroplasm, the causative agent of human babesiosis, endemic in the United States [1] and present in many other countries [2]. Besides tick transmission, *B*. *microti* is transmitted through blood transfusion [3]. It causes asymptomatic to severe illness [1]. Onset of infection is often characterized by a flu-like syndrome associated with fever, chills and headache. In few cases, the disease may evolve in an acute phase where the parasite start growing in patient blood leading to anemia and clinical complications resulting from hemolysis. Severe onset symptoms may include acute respiratory failure, organ failure and disseminated intravascular coagulation [4]. The disturbance of the coagulation system was also described during veterinary infections with other *Babesia* species, which was involving kallikrein but also non-identified factors [5, 6].

Thrombospondin type 1 repeats are protein domains that are shared between mammal host and apicomplexan parasites. In mammals, thrombospondin type 1 protein is a major component of platelet alpha granules [7, 8]. The activation of platelets leads to the display of thrombospondin on their surface [9], it mediates platelet-platelet interaction [10], as well as the interactions of platelets with other cells [11]. In apicomplexans, thrombospondin-related adhesive proteins (TRAPs) are the most well studied proteins presenting a TSP1 domain. TRAPs are micronemal and surface proteins containing a signal peptide, a von Willebrand factor A domain (VWA), a thrombospondin type 1 domain (TSP1), a transmembrane region and a short cytoplasmic tail containing a penultimate amino acid residue tryptophan (W) [12–14] which have been implicated in gliding motility [15, 16–18]. Therefore, TRAPs are essential for apicomplexan gliding motility and cell invasion [12, 14, 19]. Few other TSP1 domain-containing proteins can also be found in databases. Most of them remain uncharacterized, but all share only the small stretch of sequence homology corresponding to the TSP1 domain with host proteins.

The protective function of the immune system resides in the capacity of immune cells to discriminate between self and non-self-antigens. Molecular mimicry is the property of a given pathogen to share antigenic determinants with the host [20, 21]. Diverse pathogens produce a range of ‘mimics’ that resemble host components in both structure and function [22]. There are numerous examples of pathogens that mimic human cells including *Trypanosoma cruzi*, which mimics myocardial cells [23], *Campylobacter jejuni*, which mimics a human ganglioside [24], human cytomegalovirus mimics endothelial receptors [25] and dengue virus non-structural protein 1 (NS1) mimics coagulatory molecules [26]. Molecular mimicry is one of the parasitic strategies to escape immune defenses, its consequences are immune evasion or autoimmunity [27, 28]. Immune evasion is a common strategy for viruses, bacteria and protozoa, as well as metazoan helminths to evade host immunity [29, 30]. Molecular mimicry facilitates immune evasion because the host is averse to harming itself [31], through self-tolerance mechanisms, which eliminate autoreactive T-cells [28, 32]. These mechanisms protect the host against autoimmunity and mimics from the immune response, depending on the extent to which mimicking parasites are structurally similar to self-proteins [31]. Moreover, molecular mimicry is reported to induce an autoimmune response in the infected host [26, 33–35]. Mimicry can stimulate the destruction of host tissues, depending on the structural resemblance degree between parasite and host epitopes and/or the repeated activation of autoreactive T-cells during infection [28].

In this context, we aimed to characterize non-enzyme proteins in *B*. *microti* to search for possible molecular mimicry mechanism interacting with the coagulation system. Therefore, we focus our interest on the TSP1 domain-containing protein presenting the most similar domain with host thrombospondin. For indication of a molecular mimicry between TSP1 domains from the parasite and the host molecules, we used different measures of sequence homology analysis, tertiary structural-relationship analysis and investigation of shared epitopes. For possible safe vaccine candidate, the host immune response against the non-mimic, TSP1-free recombinant protein truncate was also evaluated.

## Materials and methods

### Parasites and experimental animals

The human isolate *B*. *microti* Gray strain (US type, American Type Culture Collection, Catalog No. 30221) was maintained in Golden Syrian hamsters by intraperitoneal injection with 1x10^7^ *B*. *microti*-infected erythrocytes. The specific pathogen-free (SPF) female Golden Syrian hamsters were purchased from Clea, Japan. Parasitemia was monitored by counting parasitized erythrocytes on Giemsa-stained thin blood smears. The blood was harvested with heparin when infected red blood cells reached at least 50% parasitemia or more. Five 6-weeks-old female ICR mice were purchased from Clea, Japan were used for the immunization experiments. All animal experiments described in this article were conducted in accordance with the Guiding Principles for the Care and Use of Research Animals promulgated by Obihiro University of Agriculture and Veterinary Medicine (Approval number for mouse experiments: <25–124>, for hamster experiments: <25–126>). Animals were housed in cages kept in shelters with 12 hours' light and dark cycle at specific pathogen free room. Animals were fed ad libitum on maintenances ration purchased from Clea, Japan. Drinking water was provided ad libitum. Room temperature was kept at 23°C. Animals were monitored twice daily. Animal health and well-being were assessed by the daily rate of food intake. To minimize animal suffering and distress, isoflurane was applied for anesthesia in rodent experiments. Animals were euthanized using overdose of isoflurane.

### Bioinformatics analysis

*B*. *microti* strain R1 genome new annotation (release April 2017) was screened for thrombospondin type 1 domain (TSP1)-containing proteins by request for annotated domain in InterPro (https://www.ebi.ac.uk/interpro/entry/IPR000884/proteins-matched?taxonomy=1133968). We used PiroplasmaDB (http://piroplasmadb.org/piro/) to visualize gene models and recover DNA and amino acid sequences. PiroplasmaDB is also a good interface to InterPro motifs predictions. *B*. *microti* RNAseq data from SRA database with accession No. ‘PRJNA218917’ to ‘PRJNA218922’ were used to validate gene expression. It should be noticed that the GenBank obsolete versions of proteins with accession numbers CCF75984.1 and CCF74291.1 were split into two CDS and the TSP1 containing peptides are now encoded by genes with new locus IDs BmR1_04g09041 and BmR1_03g00437, respectively. The protein with locus ID 'BmR1_04g09041' contained a single TSP1 domain, was selected for our study using *B*. *microti* Gray strain (S1 Fig). Presence of signal peptides were performed at SignalP (http://www.cbs.dtu.dk/services/SignalP/). Trans-membrane regions were predicted using the TOPCONS webserver [36], which uses six different trans-membrane prediction algorithms to make a consensus prediction.

The selection of gene truncates, was based on the presence or absence of the TSP1 domain, which enabled us to have one truncate having a TSP1 domain and the other not to contain a TSP1 domain. The TSP1 domain-containing truncate was designed to test mimicry through the presence of shared epitopes and the TSP1 domain-free truncate was relevant to study the immunogenicity of BmP53 and potential to be a safe vaccine candidate.

The IntFOLD pipeline [37, 38] was used to predict the 3D structure and disordered regions of *B*. *microti* P53 protein. The IntFOLD-TS [39] protocol was used to predict the 3D structure of the TSP1 domain, using the protocol described in Roche *et al*. 2016 [40]. In addition, the IntFOLD-DR protocol, which runs DISOclust [41] was used to predict the propensity of the *B*. *microti* P53 protein to contain disordered regions. TM-align [42] was used to undertake structural superposition of the domain 3D structural model of the TSP1 from *B*. *microti* P53 protein onto the crystal structure of the TSP1 domain from *Homo sapiens* [PDBID 1LSL], producing a TM-score related to the fold similarity.

Homologous proteins from other related hem protozoans and mammals were found using a BLASTp analysis at NCBI server and the degree of sequence conservation was assessed using the Clustal-W method using the MegAlign-DNASTAR program (Netwell, Japan). Furthermore, identical aa residues between equivalent TSP1 domains from the homologous proteins were compared using the GENETYX version 7.0 (Genetyx Corporation, Japan). A phylogenetic tree based on aa residues of equivalent TSP1 domains from BmP53 and homologous proteins was assessed at Phylogeny.fr server using “à la carte” options with ClustalW for the multiple alignment, no alignments curation, BioNJ for the phylogenetic analysis with the Jones-Taylor-Thornton substitution matrix and a Gamma distribution parameter of 1.

### RNA isolation and *BmP53* gene transcriptional analysis

Total RNA was prepared by lysing *B*. *microti* Gray strain-infected hamster erythrocytes with TRIzol reagent (Life Technologies, USA), followed by chloroform extraction and precipitation performed with isopropyl alcohol and ethanol. cDNA was prepared from RNA isolated from *B*. *microti* Gray strain parasites with the use of a SuperScript reverse transcriptase kit (Life Technologies, USA) following the instructions provided by the company. In RT-PCR analyses, *B*. *microti* Gray strain secreted antigen 1 [BmSA1], (Genbank accession No. 'JX112361.1') was used as a control for *B*. *microti* Gray strain parasites and its primers were according to [43]. Sequencing was undertaken with the ABI PRISM 3100 Genetic Analyzer automated sequencer (Applied Biosystems, USA) using M13 forward, reverse and internal DNA primers.

### Selection and cloning of *BmP53* gene truncates into pGEX-4T-1 vector

The selected truncates were BmP53tr1-TSP1 (619–1116 bp) and BmP53tr2 (868–1275 bp), with expected amplicon DNA sizes of 498 bp and 408 bp, respectively. For cloning, two pairs of oligonucleotide primers with *Bam*HI and *Xho*I restriction enzyme sites were designed and used for the amplification of both truncates, BmP53tr1-TSP1 (forward primer: 5′-CGGGATCCGAGAAGATAAAGGATGAA-3′; reverse primer: 5′-CGCTCGAGTTATTTATAGCTAGAGCTCAG -3′) and BmP53tr2 (forward primer: 5′-CGGGATCCAGGTTAATGAAGCTGCCA-3′; reverse primer: 5′-CGCTCGAGTTACGCAAATTGGGCAAATGA-3′). The PCR products were ligated to the *Bam*HI and *Xho*I restriction sites of the glutathione S-transferase (GST) fusion expression vector, pGEX-4T-1 (GE Healthcare Life Sciences, UK). The constructs of the resulting plasmids were checked for accurate insertion by digestion with *Bam*HI and *Xho*I according to the manufacturer’s instructions (ROCHE Diagnostics, Germany).

### Expression and purification of recombinant *BmP53* selected truncates

The cloned nucleotide sequences were expressed as glutathione S-transferase (GST) fusion proteins in the *E*. *coli* BL21 strain according to the manufacturer’s instructions (GE Healthcare Life Sciences, UK). The expression was done at 37°C for 4 hours after induction with 0.5 mM isopropyl β-D-1-thiogalactopyranoside (IPTG). The resulting *E*. *coli* cells were washed three times with phosphate-buffered saline (PBS) pH 7.4, lysed with 1% Triton in PBS, sonicated and then centrifuged at 10,000 rpm for 30 min at 4°C. Supernatant was purified with Glutathione-Sepharose 4B beads according to the manufacturer’s instructions (GE Healthcare Life Sciences, UK). The protein concentration was measured by using a Pierce BCA Protein Assay Kit (Thermo Scientific, USA).

### Generation of mouse anti-BmP53 antibodies

A total of six-weeks old female ICR mice (Clea, Japan) were intraperitoneally immunized with 100 μg of purified soluble rGST-BmP53tr1-TSP1 emulsified with an equal volume of complete Freund’s adjuvant (Difco Laboratories, USA). Two additional boosters consisting of 50 μg of rGST-BmP53tr1-TSP1 emulsified with incomplete Freund’s adjuvant (Difco Laboratories, USA) were intraperitoneally administered at 2-week intervals. The mice were bled 16 days after the last booster, and serum samples were stored at -30°C. Rabbit anti-BmSA1 sera were prepared according to the method described by Lou and co-workers [43].

### Sodium dodecyl sulfate polyacrylamide gel electrophoresis (SDS-PAGE) and Western blot analysis

To identify the native BmP53 protein in *B*. *microti*, the parasite-infected and non-infected hamster erythrocytes were analyzed using SDS-PAGE (8%) and Western blot analysis under conditions previously described by Terkawi and colleagues [44]. Briefly, *B*. *microti*-infected and non-infected erythrocytes were washed with cold PBS and then lysed in distilled water to produce a 25-fold dilution (v:v). The pellets containing parasites were washed four times with cold PBS, disrupted three times by a freeze-thaw cycle in liquid nitrogen, and then sonicated in ice slurry. The lysates were loaded to 8% SDS-PAGE gel and then blotted to nitrocellulose membranes. The blotted membranes were blocked with 0.05% Tween 20 in PBS (PBS-T) plus 5% skimmed milk and then probed with mouse anti-rGST-BmP53tr1-TSP1 and mouse pre-immune sera (1:100). A secondary antibody (1:4,000) of horseradish peroxidase-conjugated goat anti-mouse IgG (Bethyl Laboratories, Montgomery, TX, USA) was used to identify the bound proteins on the blots. Finally, the positive bands were visualized using a solution containing 3- diaminobenzidine tetrahydrochloride (DAB) and H2O2 (Dojindo, Japan). Additionally, the expressed recombinant proteins were verified by SDS-PAGE with subsequent Coomassie blue staining. Furthermore, to determine the antibody response against the non-mimic TSP1 domain-free truncate (rGST-BmP53tr2). Both rGST-BmP53tr2 and rGST were analyzed by SDS–PAGE (12%). Western blot analysis was as described above using a *B*. *microti* Gray strain experimentally infected and non-infected hamster sera, and HRP-conjugated goat anti-hamster IgG (Bethyl Laboratories, USA).

### Isolation of hamster platelets

Hamster platelets were isolated from a non-infected SPF hamster according to the method previously described by Chen *et al*. [45]. In brief, hamster whole blood containing the anticoagulant ACD (29.9 mM sodium citrate, 113.8 mM glucose, 72.6 mM sodium chloride and 2.9 mM citric acid, pH 6.4) was centrifuged at 1000×g for 10 min at room temperature. The upper layer of centrifuged lysate, which contains platelet-rich plasma was removed and placed in a 15-ml tube, then washed twice in EDTA/PBS. The washed platelets were suspended in Tyrode's solution (137 mM NaCl, 20 mM HEPES, 3.3 mM NaH2PO4, 2.7 mM KCl, 1 mg/ml BSA and 5.6 mM glucose, pH 7.4) at a concentration of 108 platelets/ml.

### Immunofluorescence Antibody Assay (IFA)

The cellular localization of BmP53 was determined by an Immunofluorescence antibody assay (IFA) according to [14]. Briefly, thin blood smears of *B*. *microti*-infected erythrocytes or non-infected isolated hamster platelets were fixed in a solution of 95% methanol and 5% acetone (v:v) at -20°C for 30 min and then probed with mouse anti-rGST-BmP53tr1-TSP1 immune sera diluted in 4% fetal bovine serum in PBS (1:100) for 1 h at 37°C in a moist chamber. A secondary antibody of goat anti-mouse or -Rabbit IgG (H+L) (Alexa-Fluor 488 conjugate) (green dye) and goat anti-mouse IgG (H+L) (Alexa-Fluor 594-conjugate) (red dye) (Life Technologies, USA) diluted in the same buffer (1:250) was applied on the smears and then incubated for 1 h at 37°C. DNA was stained with Hoechst blue dye (Dojindo Molecular Technologies, Inc., USA) in PBS (1:250) for 10 min at RT. After washing with PBS-T, the smears were mounted using a fluorescent mounting medium (Dako, USA) and then covered with a glass coverslip. Imaging was performed using All-in-One Fluorescence Microscope, BZ-X700 series (KEYENCE corporation, Japan). For the co-localization study, anti-rBmSA1 rabbit serum [43] was used as a specific marker for *B*. *microti* Gray strain parasites.

### Nucleotide sequence accession number

The partial sequence of the *B*. *microti* Gray strain BmP53 CDS overlapping both BmP53tr1-TSP1 and BmP53tr2 has been submitted to the GenBank with accession No. ‘KX174293’. Full sequence of *B*. *microti* R1 BmP53 protein is available at Accession ID SIO73859.

## Results

### Identification and characterization of *B*. *microti BmP53* gene

**Fig 1:**
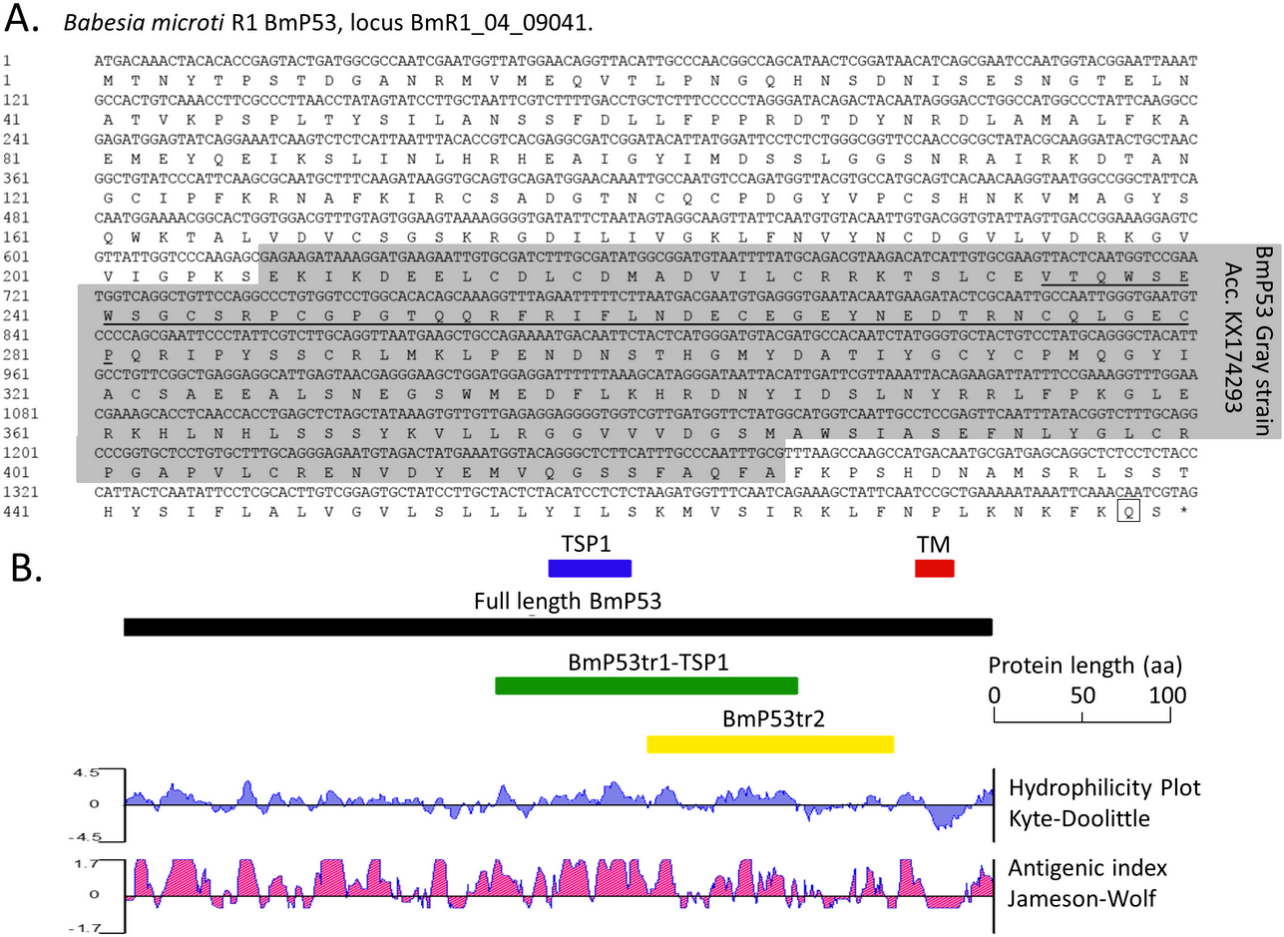
Bioinformatics analysis of the BmP53 sequence. **(A)** The DNA and translated amino acid sequences of BmP53 are shown, with the underlined segment of the sequence representing the TSP1 domain. The CDS was from genomic region LN871599 join(1510300..1511012, 1511060..1511239, 1511261..1511438, 1511689..1511869, 1511945..1512132). The consensus sequence deduced from sequencing of the PCR amplicons from the Gray strain DNA is shaded in grey (accession number KX174293). The penultimate glutamine (Q) residue is framed. **(B)** Overview of the domain structure for BmP53, showing elected truncates, in addition to hydrophilicity and antigenicity indices.

*B microti* genome encodes for four TSP1 domains containing proteins according to InterPro database. Three proteins had TSP1 domains that were present either in Pfam or SMART databases (UNIPROT accession A0A1N6LYA1_BABMR, A0A1R4AB65_BABMR and I7JDM5_BABMR and corresponding loci BmR1_04g09041, BmR1_03g00437 and BmR1_04g08640, S1 Fig). A fourth protein had more distant TSP1 domains that were neither detected by Pfam nor SMART search engines (A0A1R4AB49_BABMR, locus BmR1_03g00451, S1 Fig). This gene was unique to piroplasmida. BmR1_03g00437 and BmR1_04g08640 corresponding genes had orthologues in *Plasmodium falciparum* (loci PF3D7_0822700 and PF3D7_0315200 respectively). The gene at locus ID BMR1_04g09041 was part of a specific orthology group where *Plasmodium* species were absent (OrthoMCL: OG5_171221). The encoded protein raised our interest because it had a single TSP1 domain which was selected for our study (S1 Fig). The gene had four introns and mature mRNA consists of a CDS of 1,437 bp, encoding 479 amino acids, with a predicted 53.7-kDa molecular weight, we named this protein BmP53 (Fig 1A). No signal peptide was predicted, but BmP53 presents a single transmembrane (TM) domain (residues 437–459) with a short cytoplasmic tail (S1 Fig).

BmP53 domain organization was very different from TRAP proteins described so far in *P*. *falciparum* (S2 Fig). Localization and function might be significantly different from these proteins. Accordingly, the penultimate amino acid residue is a glutamine (Q) and not a tryptophan (W), as seen in the family of TRAP proteins. BmP53 orthologues from *Theileria orientalis* and *T*. *annulata* were sharing the same BmP53 structural architectures (No signal peptide, single TSP1 domain and a transmembrane domain with a glutamine (Q) as a penultimate amino acid). Incomplete proteins were found in other piroplasmida genomes suggesting a possible incomplete annotation of the gene in these organisms because of the number of introns. The N-terminus part of the protein upstream of the TM was hydrophilic and with good antigenicity index on analysis using the DNASTAR program (Netwell, Japan) (Fig 1B). BLASTp BmP53 homologous proteins were found among mammals but only in region of the TSP1 domain. These two regions were chosen for further immunological characterization in the parasite and host and will be amplified as truncates.

### Transcriptional analysis and expression of recombinant protein

The transcriptional expression of the BmP53 gene was among the top half most expressed genes, in the third quartile interval with relatively constant expression between the different sequenced isolates [46]. Expression of *BmP53* gene in *Babesia microti* Gray strain asexual stages was confirmed by RT-PCR analyses using BmSA1 as a control. Amplicons were obtained for the two regions of interest for which we wanted to study immunological properties (S3A Fig). Both amplified BmP53 protein truncates were sequenced and aligned with the corresponding sequences from *B*. *microti* strain R1 databases on GenBank, with 100% identity. Peptides were expressed as GST-fusion in *E*. *coli* BL21 using pGEX-4T-1 vector. Expression of recombinant proteins resulted in a purified soluble 47.3-kDa rGST-BmP53tr1-TSP1 protein and non-purified insoluble 43.4-kDa rGST-BmP53tr2 protein (S3B Fig).

### Characterization of the native *B*. *microti* Gray strain BmP53 protein

**Fig 2:**
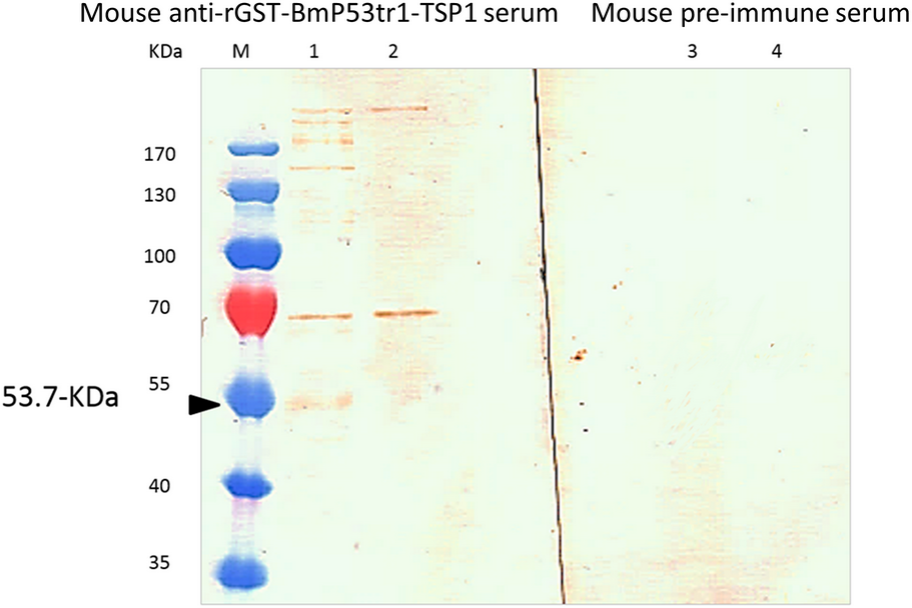
Western blot analysis to detect native BmP53 within the lysate of parasitized host erythrocytes. Lane M: Pre-stained protein marker. Lanes 1 and 3: Lysates of *B*. *microti* infected hamster erythrocytes. Lanes 2 and 4: Lysates of non-infected hamster erythrocytes. Lanes 1 and 2 were probed with mouse anti-rGST-BmP53tr1-TSP1 serum. Lanes 3 and 4 were probed with mouse pre-immune sera. Black arrow point to specific 53.7-kDa band for native BmP53.

**Fig 3:**
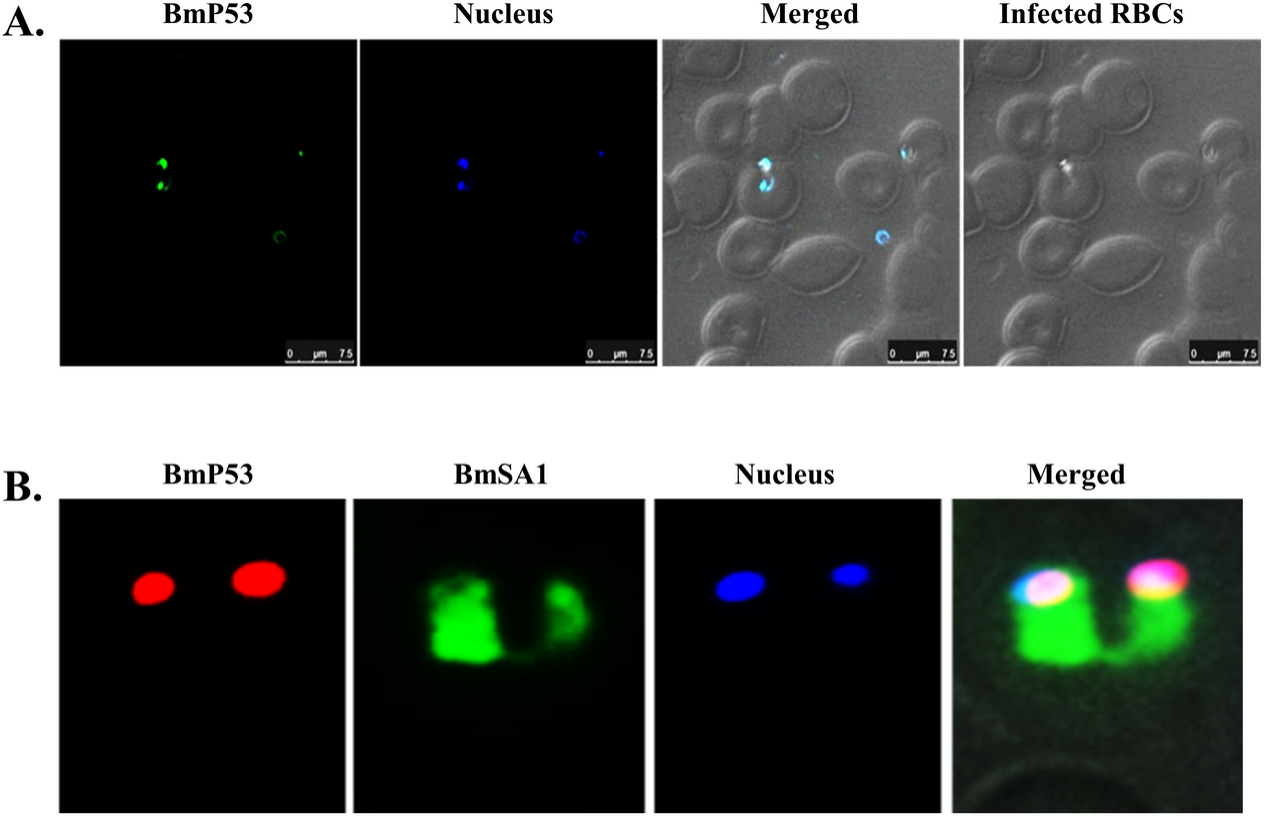
Cellular localization of the native BmP53 protein via Immunofluorescence Antibody Assay (IFA). **(A)** Thin blood smear of *B*. *microti*-parasitized hamster erythrocytes showing reactivity of parasite erythrocytic and extra-erythrocytic stages with mouse anti-rGST-BmP53tr1-TSP1 serum (green), nuclear staining (blue). Scale bar: 7.5µm. **(B)** A Co-localization study of BmP53 and BmSA1 on thin blood smear of *B*. *microti*-parasitized hamster erythrocytes showing reactivity of parasite erythrocytic stage with mouse anti-rGST-BmP53tr1-TSP1 serum (red), rabbit anti-BmSA1 serum (green), nuclear staining (blue).

The native BmP53 protein was characterized by Western blot analysis and Immunofluorescence Antibody Assay (IFA) using mouse anti-rGST-BmP53tr1-TSP1 serum. In the Western blot analysis, lysates of *B*. *microti*-infected and non-infected hamster erythrocytes were probed with mouse anti-rGST-BmP53tr1-TSP1 serum and pre-immune serum, respectively. A specific band of approximately 53.7-kDa was detected only from lysates of *B*. *microti*-infected erythrocytes when probed with mouse anti-rGST-BmP53tr1-TSP1 serum. There are also three additional specific bands with higher molecular weights. We also detected from the same lysates, one band between 130- and 170-kDa and two bands over 170-kDa (Fig 2). Furthermore, Immunofluorescence Antibody Assay (IFA) on thin blood smears of *B*. *microti*-infected hamster erythrocytes stained with immune sera to rGST-BmP53tr1-TSP1, show reactivity with intracellular and extracellular parasites (Fig 3A). In addition, a co-localization study of BmP53 and BmSA1 showed the same reactivity (Fig 3B). From both IFA observations, mouse anti-serum reacted with BmP53 to yield fluorescence in the parasite cytoplasm with non-secretory properties. These findings suggest that the antiserum against rGST-BmP53tr1-TSP1 could detect the endogenous BmP53 protein in the parasite.

### Antibodies directed to BmP53 TSP1domain cross react with isolated host platelets

**Fig 4:**
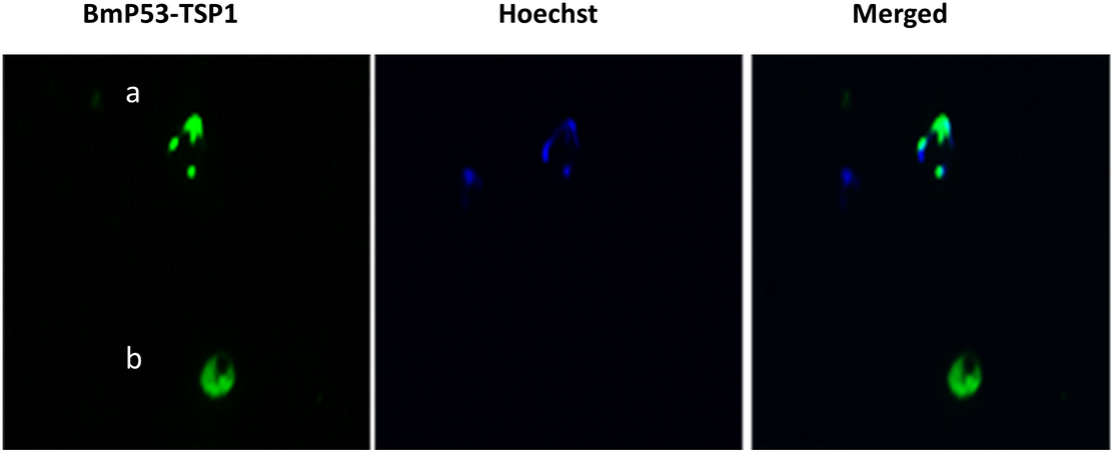
Immunofluorescence Antibody Assay (IFA) showing non-nucleated cross-reactant hamster cells. Thin blood smear of *B*. *microti*-infected hamster erythrocytes showing reactivity of mouse anti-rGST-BmP53tr1-TSP1 serum (green) with the parasite (a) and non-nucleated hamster cells (b). Nuclear staining was performed with Hoechst DNA specific dye (blue).

**Fig 5:**
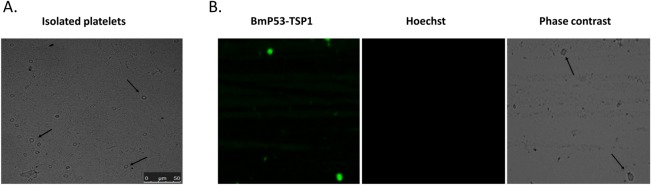
Detection of cross-reacting shared epitopes between BmP53 and hamster platelets. **(A)** Phase contrast image of the thin smear of isolated platelets from whole blood of non-infected SPF hamster. Black arrows indicate platelets. Scale bar: 50µm. **(B)** Immunofluorescence antibody assay (IFA) on isolated platelets from non-infected blood of SPF hamster, showing specific reactivity of platelets surface proteins with mouse anti-rGST-BmP53tr1-TSP1 (green), nuclear staining (blue).

Immunofluorescence Antibody Assay (IFA) on thin blood smears of *B*. *microti*-infected hamster erythrocytes stained with immune sera to rGST-BmP53tr1-TSP1, revealed the presence of a small number of non-nucleated reactant hamster cells (Fig 4). Hence, it was crucial to further describe these cross-reacting cells in the absence of infection to sidestep any parasitic reactants. We surmised that the cells were platelets from their morphology and knowledge from the literature. Subsequently, we isolated platelets from whole blood of non-infected SPF hamster. The isolated platelets were presented with their characteristic morphology when observed in thin direct smears by phase contrast image using an All-in-One Fluorescence Microscope, BZ-X700 series (KEYENCE corporation, Japan) (Fig 5A). On the application of IFA on thin direct smears of isolated platelets stained with immune sera to rGST-BmP53tr1-TSP1, we detected the cellular localization of a cross-reacting protein on the surface of isolated hamster platelets (Fig 5B), suggesting that both the *B*. *microti* P53 TSP1 domain and the hamster platelet proteins share similar epitopes. Therefore, the anti-rGST-BmP53tr1-TSP1 antibodies could be a marker for hamster platelets. Results suggest the presence of a molecular mimicry between BmP53 protein and host molecules, which can be classified as a low extent of mimicry because it might be restricted to a TSP1 domain not to the full BmP53 protein sequence.

### Phylogenetic analysis of host and *B*. *microti* BmP53 TSP1 domain

**Fig 6:**
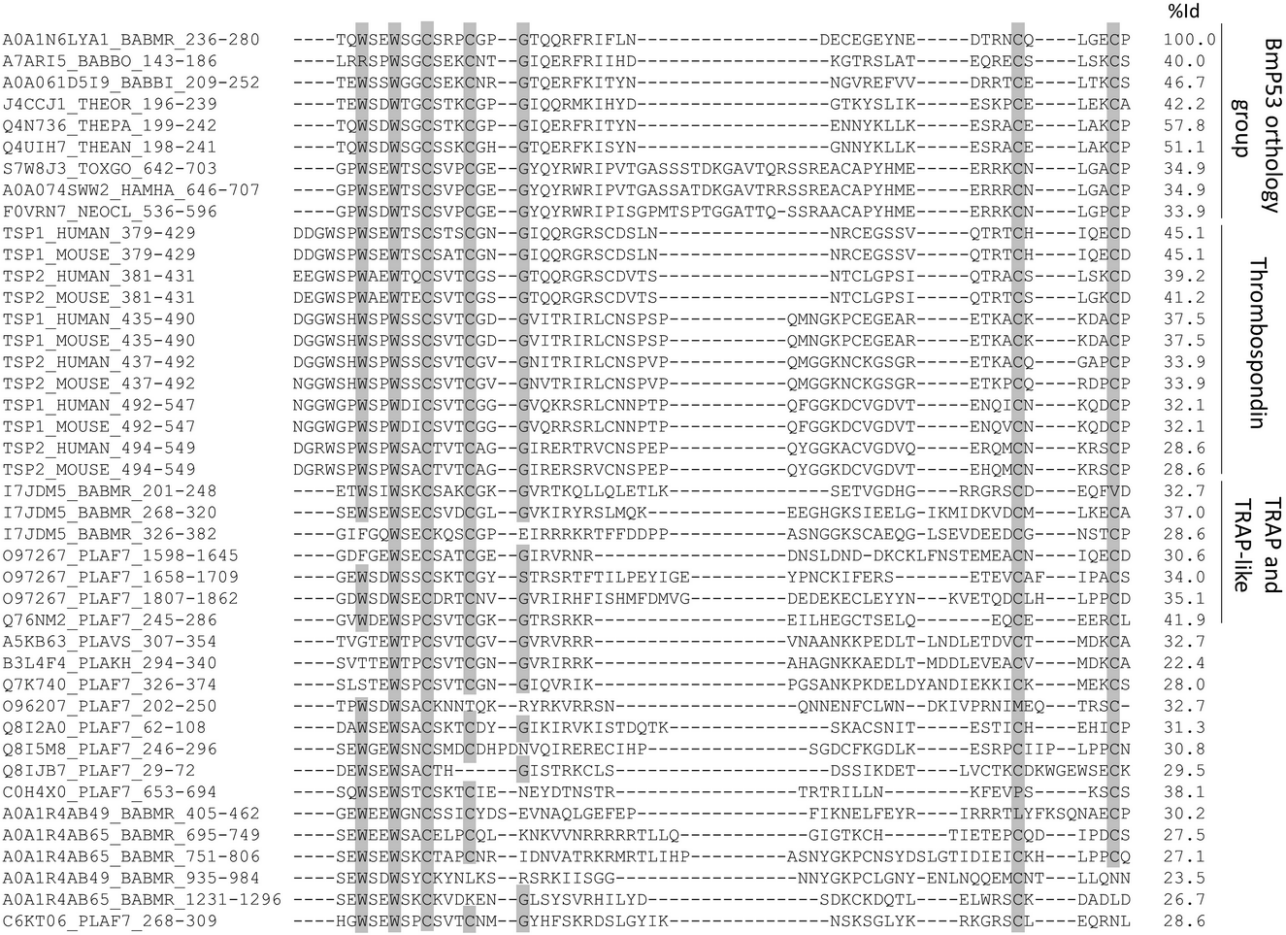
Multiple sequence alignment of TSP1 domains from apicomplexan proteins and human or mouse thrombospondin. Domains are labelled by the Uniprot ID of the protein and coordinates in the amino acid sequence. The percent of sequence identity with *B*. *microti* BmP53-TSP1 domain is given for each TSP1 domains and residues conserved in more than 80% of the sequences in the alignment were highlighted in grey.

**Fig 7:**
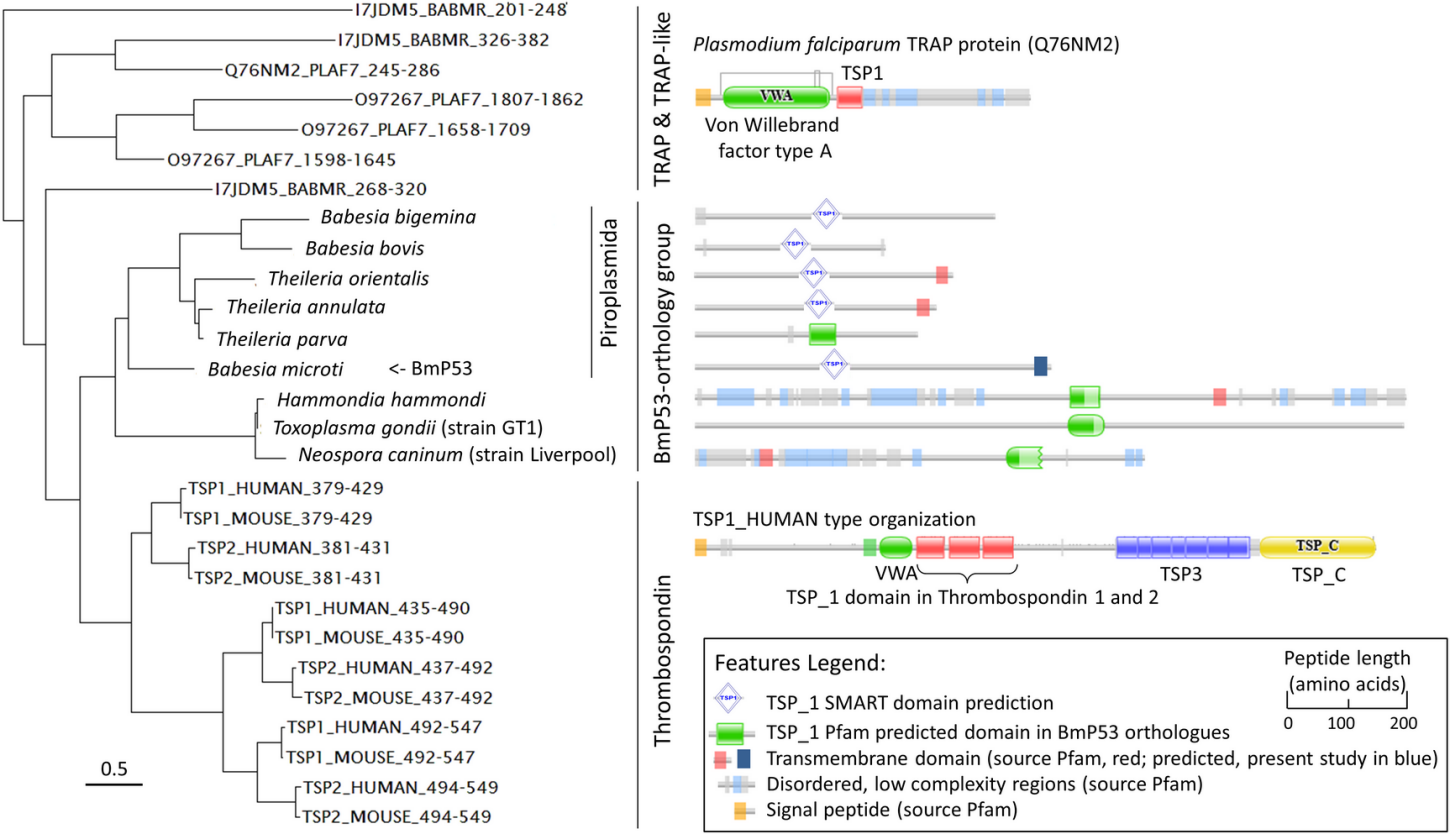
Close phylogenetic relationship of TSP1 domains from *B*. *microti* P53 and its orthologues with Thrombospondin TSP1 domains. Comparison is performed with TSP1 domains from TRAP and TRAP-like proteins of *B*. *microti* and *P*. *falciparum*. Phylogenetic analysis was performed using the Phylogeny.fr server using “à la carte” options, with clustal W for the multiple alignment, BioNJ for the phylogenetic analysis with a Gamma parameter of 1 and the Jones-Taylor-Thornton substation matrix. TSP1 domains of thrombospondin and TRAP protein are identified by their coordinates and UNIPROT ID. UNIPROT species ID: BABMR, *Babesia microti*; PLAF7, *Plasmodium falciparum* (isolate 3D7); HUMAN, *Homo sapiens* and MOUSE, *Mus musculus*. The scale bottom left-hand corner indicates the number of substitutions between sequences. The scale in the legend indicate the length of the protein that are schematically represented on the right-hand part of the figure.

The BmP53 TSP1 domain was highly conserved among homologues in apicomplexan species and thrombosporin-1 from mammals (Fig 6). The amino acid sequence spanning the BmP53 TSP1 domain was more identical to those of *Homo sapiens* and *Mus musculus*, than many TSP1 domains from other protein of apicomplexa (Fig 1). Notably, this high percent of identity, which was calculated on TSP1 domain aa residues was not correlated with protein organization or sequence homologies at the level of the full-length polypeptides (Fig 7). The TSP1 domain from BmP53 orthologues forms a specific and very stable phylogenetic cluster (S4 Fig). Uniqueness of BmP53 TSP1 domain was increased by the fact that Pfam database did not identified the TSP1 domain in several BmP53 orthologues (Fig 7), whether SMART search engine better do it. The closeness of BmP53 TSP1 domain sequence with mammalian Thrombospondin TSP1 domains, when compared to other TSP1 domains present in *B*. *microti*, was confirmed by phylogeny (S5 Fig). The closeness was also observed when BmP53 sequence is compared with *B*. *microti* or *P*. *falciparum* TRAP proteins (Fig 7). The BmP53 cluster organization was very consistent with the species tree of apicomplexan parasites [47] beside the absence of orthologues in *Plasmodium* species. These observations suggest a specific evolution of the BmP53 TSP1 domain which makes sequence similarities closer to the mammalian thrombospondin TSP1 domains.

#### Structural relationship between TSP1 domains from *B*. *microti* and host

**Fig 8:**
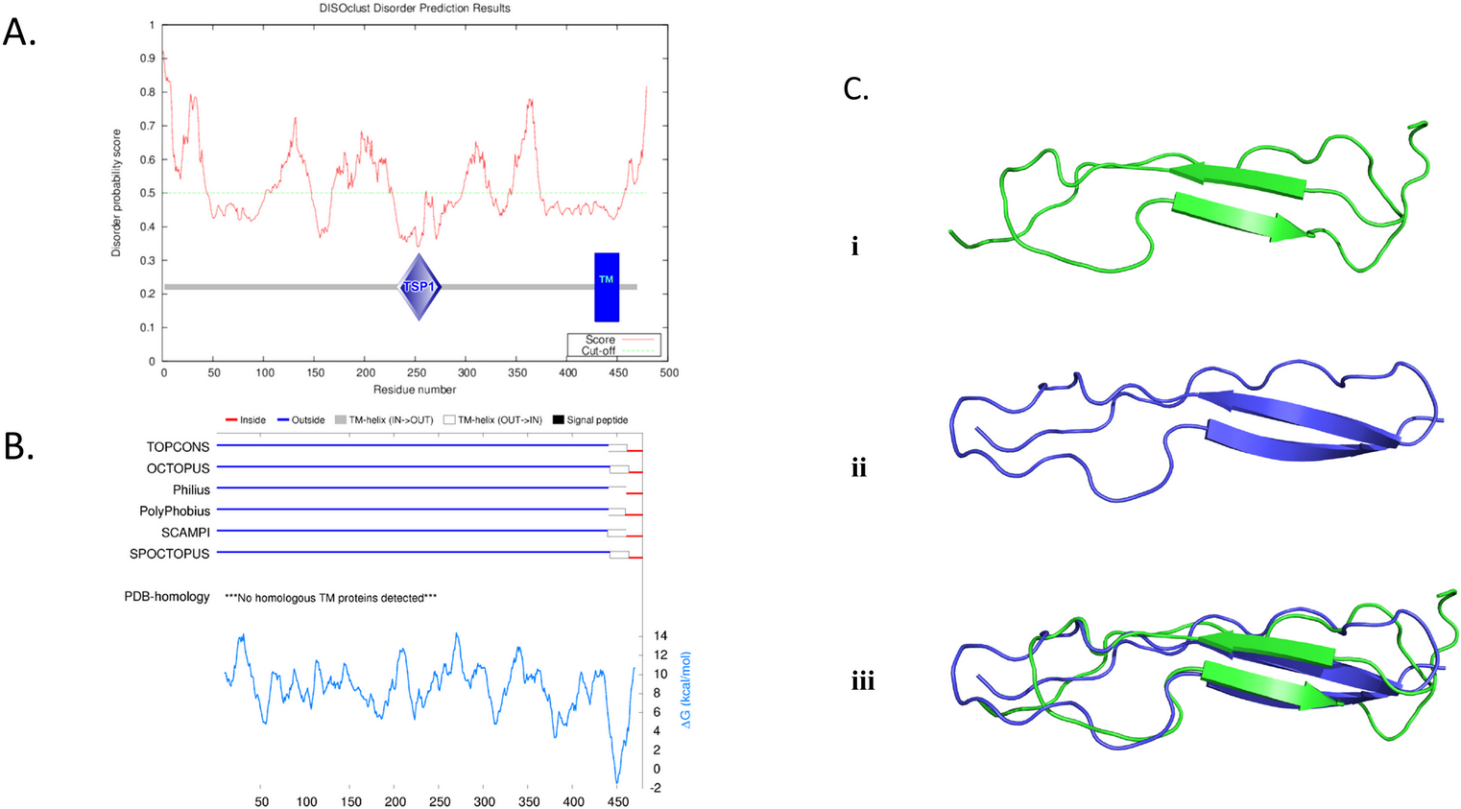
Disorder regions and trans-membrane domain predictions for BmP53 and TSP1 domain structure predictions. **(A)** Disorder regions prediction for BmP53. DISOclust protein disorder prediction is shown. The SMART domain definitions are superimposed to highlight the TSP1 and Trans-membrane domain locations. **(B)** Trans-membrane domain prediction for BmP53 using TOPCONS server. **(C)** Comparing TSP1 domain structure from *B*. *microti* P53 and *Homo sapiens*. [PDBID 1LSL]. (i) Model of the TSP1 domain from the BmP53 protein (green), built using the IntFOLD server. (ii) Crystal structure of the Thrombospondin-1 Type 1 (TSP1-1) domain from *Homo Sapiens* [PDBID 1LSL] (Blue). (iii) Superposition of TSP1 domains from BmP53 protein (green) and PDBID 1LSL (blue), which have a TM-score of 0.5936, thus both structures have the same fold.

The BmP53 protein was shown to contain disordered regions using the IntFOLD-DR protocol which runs DISOclust [41]. SMART predictions confirmed that BmP53 contains two domains outside the two large disordered regions: a TSP1 domain and a helical trans-membrane domain (Fig 8A). The presence of the helical trans-membrane domain close to the C-terminal of the protein is confirmed by a meta-method combining six trans-membrane predictors (Fig 8B). The predicted 3D structure of the TSP1 domain from BmP53 protein, built using IntFOLD-TS, has the same fold as the crystal structure of Thrombospondin-1 Type 1 (TSP1-1) domain from *Homo Sapiens* [PDBID 1LSL], although the beta-sheets which are slightly shorter (Fig 8C). Structure similarities were confirmed using TM-align giving a TM-score of 0.5936. TM-scores ranging from 0.4 to 0.6 have been shown to correspond to related folds [48].

### Evaluation of host immune response against BmP53 non-mimic parts

**Fig 9:**
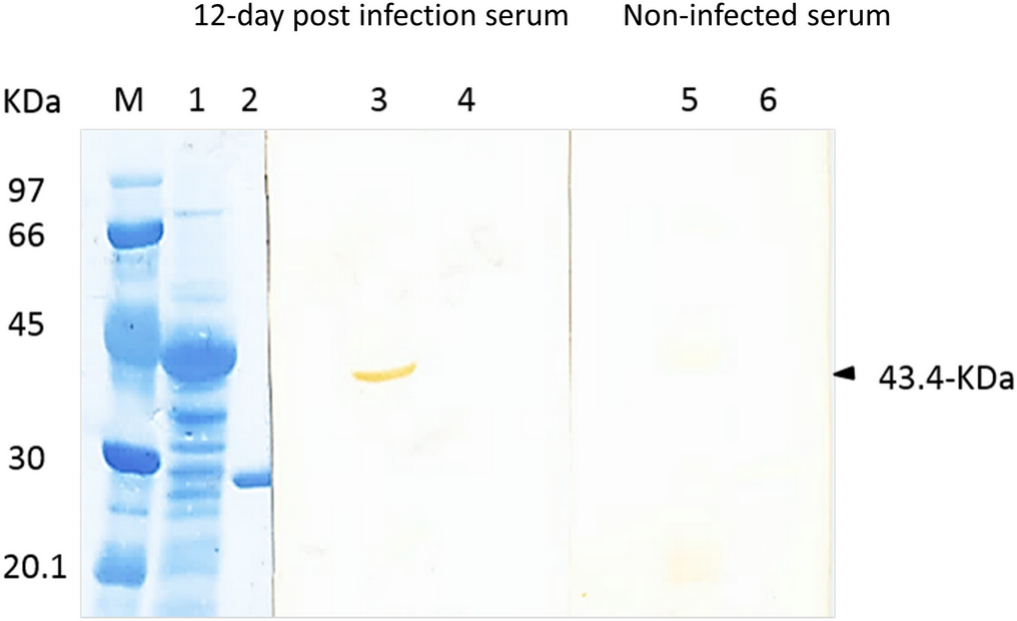
Evaluation of host immune response against the non-mimic, TSP1 domain-free truncate (rGST-BmP53tr2) using SDS-PAGE and Western blot analysis. Lane M: Low molecular weight marker. Lanes 1, 3 and 5: *E*. *coli* pellet contained rGST-BmP53tr2. Lanes 2, 4 and 6: Purified rGST. Lanes M, 1 and 2 were stained by amide black stain. Lanes 3 and 4 were probed with experimentally infected hamster serum (12-day post infection). Lanes 5 and 6 were probed with non-infected hamster serum. Black arrow point to a 43.4-kDa band revealing the presence of host immune reactivity against rGST-BmP53tr2.

Host immune reactivity against the insoluble expressed non-mimic, TSP1 domain-free truncate (rGST-BmP53tr2) was tested by Western blot analysis. In Western blot analysis, the *E*. *coli* pellet containing the non-purified insoluble expressed rGST-BmP53tr2 and purified rGST were probed with a *B*. *microti* Gray strain experimentally infected hamster serum (12-day post infection) and a non-infected serum from SPF hamster. The infected hamster serum was able to detect the rGST-BmP53tr2 (43.4-kDa) only but not rGST protein (Fig 9). Inferring that, a notable host immune reactivity against rGST-BmP53tr2 was detected with infected sera.

## Discussion

BmP53 has a unique organization. *B*. *microti* strain R1 proteome was screened for thrombospondin type 1 domain-containing proteins (TSP1 domain). The protein with locus ID 'BBM_04g09041' called BmP53 was selected because it contained a single TSP1 domain and no signal peptide (Fig 7). BmP53 protein was conserved among apicomplexan, while *Plasmodium falciparum* showed no orthologues. Protein organization was different from TRAP proteins studied in the malaria parasite (Fig 7 and S2 Fig), which increased our interest. The percent of identity between BmP53 TSP1 domain and mammalian thrombospondin TSP1 domain was also remarkably high for BmP53 and we therefore continue on immunological studies.

Analysis of BmP53 protein architecture showed no signal peptide, a single TSP1 domain, a transmembrane domain (TM) and a short cytoplasmic tail with a penultimate glutamine amino acid residue (Q). Therefore, BmP53 structural architecture is different from the well characterized TRAP and TREP (S2 Fig). The *P*. *falciparum* TRAP TSP1 domain-proteins containing a von Willebrand factor A domain (VWA). The apicomplexan TRAP proteins are micronemal and surface proteins containing a signal peptide, a transmembrane region and a short cytoplasmic tail. Protein length and structure is also far from other TRAPs which having non-typical architectures [12–14]. For example, The TRAP-related proteins in *P*. *berghei* (TREP) [15] and *P*. *vivax* (TRAMP) [49] are missing the VWA domains. BmP53 organization differs also from sporozoite proteins CS and S21.

BmP53 TSP1 domain was phylogenetical related to the first TSP1 domains of the thrombospondin proteins of mouse and human and was part of a very specific orthology group (Fig 7). The protein organization in piroplasmida of the BmP53 orthology group were very consistent with the presence of single TSP1 domain and one transmembrane domain. A review of the gene models in *Theileria parva*, *Babesia bigemina* and *B*. *bovis* will be necessary to validate their N- and C-terminal ends. Protein organization in coccidian homologues was in agreement with the idea that they could be orthologues despite the considerable increase in length. Homology between parasite and host TSP1 domain was also found at the structural level (Fig 8). All similarities were restricted to the TSP1 domain which confirm that BmP53 is not the result of a lateral gene transfer from host as shown for the lactate dehydrogenase gene [47]. Unfortunately, sequence homologies and protein description does not help us to better characterize the cellular function of BmP53 protein.

Anti-rGST-BmP53tr1-TSP1 polyclonal sera were prepared and used to identify native BmP53 in the *B*. *microti* parasites by Western blot analysis (WB) and immunofluorescence assay (IFA). A specific 53.7-kDa band was detected in *B*. *microti* lysates by WB (Fig 2). Additionally, three non-specific bands with higher molecular weights were detected from the same lysates of *B*. *microti*-infected erythrocytes, one band between 130- and 170-kDa, while, the other two bands over 170-kDa, which may be from cross-reaction with other *B*. *microti* TSP1 domain-containing proteins. Evaluation of the cellular location of BmP53 in the parasite by IFA besides the protein architecture description analysis results, revealed that BmP53 was a non-secretory membranous protein. Cross-reactivity with host platelets suggested molecular mimicry of BmP53. Molecular mimicry is the property of a given pathogen to share antigenic determinants with the host [20, 21]. Accordingly, we might consider that mimicry may only be useful for the parasite extra-erythrocytic stages when the BmP53 is exposed to the blood circulation. Molecular mimicry was never described for TSP1 domains found in other proteins. The mimicry property might therefore be restricted to members of the BmP53 orthology group. Therefore, the functional impact of the molecular mimicry between TSP1 domains from *B*. *microti* BmP53 and host platelets proteins has to be evaluated by other studies.

Interestingly, cross-reactivity with human platelets was also reported with antibodies directed against dengue virus nonstructural protein 1 (NS1) which mimics host molecules [26], and many blood disorders were reported during *Babesia* infections [4–6]. We report the mouse anti- BmP53-TSP1 antibodies as markers for hamster platelets. We imagine that BmP53 antibodies might help studying the pathogenesis of blood disorders induced by piroplasmida in mammals. As an example, idiopathic thrombocytopenic purpura was induced in mice by intraperitoneal injection of antiplatelet antibody [50]. Further applications might resulted from the host immune response against the non-mimic, TSP1 domain-free truncate (rGST-BmP53tr2). A specific host immune reactivity against the rGST-BmP53tr2 was noticed during infection (Fig 9). Thus, it may be a safe candidate for future vaccine studies.

## Supporting information

S1 Fig Domain architectures of *B. microti* proteins containing TSP1 domains. Schematic drawing showing the domain architectures of the *B*. *microti* was recovered from Pfam database. Additional features were predicted using specific web servers. Gene and proteins were identified according to their locus and UNIPROT ID respectively.
(TIF)

S2 Fig Structure of TSP1 domain containing protein in *Plasmodium falciparum*. Structural description was recovered at Pfam database. Additional predictions were recovered from InterPro database.
(TIF)

S3 Fig Gene transcriptional analysis and protein expression of selected *B. microti* Gray strain P53 truncates. **(A)** Transcriptional analysis of *B*. *microti* P53 gene truncates by RT-PCR. Lane M: DNA ladder, lane 1: BmP53tr1-TSP1 mRNA, 498 bp, lane 2: BmP53tr2 mRNA, 408 bp and lane 3: BmSA1, 909 bp. **(B)** SDS-PAGE of expressed GST-fused recombinant BmP53 truncates stained by Coomassie blue stain. Lane M: Low molecular weight marker. Lanes 1, 2 and 3: Different elutes contain purified soluble expressed GST-rBmP53tr1-TSP1 from lysed bacteria supernatant, 47.3-KDa. Lane 4: Insoluble expressed GST-rBmP53tr2 protein in E. coli pellet, 43.4-KDa. Lanes 5, 6 and 7: Different elutes missing the non-purified insoluble expressed GST-rBmP53tr2.
(TIF)

S4 Fig TSP1 domain from BmP53 orthologues are grouped in a single and specific phylogenetic cluster. TSP1 domains are identified by their coordinates and proteins by their UNIPROT ID and UNIPROT species ID: BABBI, *Babesia bigemina*; BABBO: *Babesia bovis*; BABMR, *Babesia microti*; HAMHA, *Hammondia hammondi*; NEOCL, *Neospora caninum*; PLAF7, *Plasmodium falciparum* (isolate 3D7); PLAKH, *Plasmodium knowlesi* (strain H); PLAVS, *Plasmodium vivax* (strain Salvador I); THEAN, *Theileria annulata*; THEOR, *Theileria orientalis* (strain Shintoku); THEPA, *Theileria parva*; TOXGO, *Toxoplasma gondii* (isolate GT1). The scale top right-hand corner of the tree indicates the number of substitutions between sequences.
(TIF)

S5 Fig Phylogenetic relationship between *Babesia microti* TSP1 domains and TSP1 domains found in best homologues. TSP1 domains are identified by their coordinates and proteins by their UNIPROT ID and UNIPROT species ID: BABBI, *Babesia bigemina*; BABBO: *Babesia bovis*; BABMR, *Babesia microti*; HAMHA, *Hammondia hammondi*; NEOCL, *Neospora caninum*; THEAN, *Theileria annulata*; THEOR, *Theileria orientalis* (strain Shintoku); THEPA, *Theileria parva*; TOXGO, *Toxoplasma gondii* (isolates GT1). The scale top right-hand corner of the tree indicates the number of substitutions between sequences.
(TIF)
