# Correction: Human babesiosis: Indication of a molecular mimicry between thrombospondin domains from a novel *Babesia microti* BmP53 protein and host platelets molecules

**DOI:** 10.1371/journal.pone.0189383

**Published:** 2017-12-05

**Authors:** Ahmed Abdelmoniem Mousa, Daniel Barry Roche, Mohamad Alaa Terkawi, Kyohko Kameyama, Ketsarin Kamyingkird, Patrick Vudriko, Akram Salama, Shinuo Cao, Sahar Orabi, Hanem Khalifa, Mohamed Ahmed, Mabrouk Attia, Ahmed Elkirdasy, Yoshifumi Nishikawa, Xuenan Xuan, Emmanuel Cornillot

The affiliation of the third author is incorrect. Mohamad Alaa Terkawi is not affiliated with #1 but with #2 National Research Center for Protozoan Diseases, Obihiro University of Agriculture and Veterinary Medicine, Obihiro, Hokkaido, Japan.

The affiliation of the fourteenth author is incorrect. Yoshifumi Nishikawa is not affiliated with #3 but with #2 National Research Center for Protozoan Diseases, Obihiro University of Agriculture and Veterinary Medicine, Obihiro, Hokkaido, Japan.

The affiliation of the fifteenth author is incorrect. Xuenan Xuan is not affiliated with #3 but with #2 National Research Center for Protozoan Diseases, Obihiro University of Agriculture and Veterinary Medicine, Obihiro, Hokkaido, Japan.
