# Supplementary figures and images for: Human babesiosis: Indication of a molecular mimicry between thrombospondin domains from a novel Babesia microti BmP53 protein and host platelets molecules
Source: PLoS One. 2017 Oct 17;12(10):e0185372. doi: 10.1371/journal.pone.0185372 (PMC5644982; doi:10.1371/journal.pone.0185372)

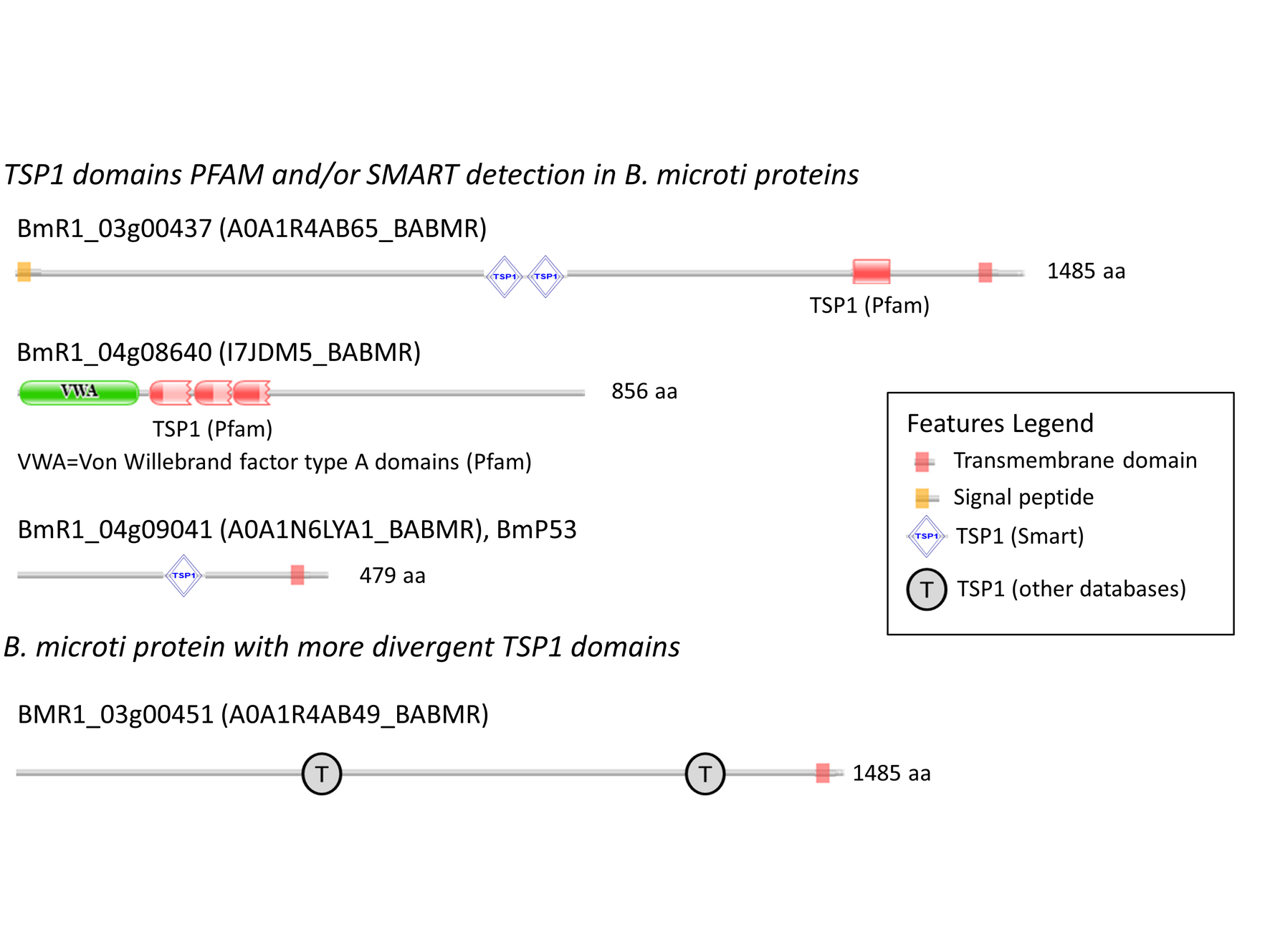

Supplement: S1 Fig — Schematic drawing showing the domain architectures of the B. microti was recovered from Pfam database. Additional features were predicted using specific web servers. Gene and proteins were identified according to their locus and UNIPROT ID respectively. (TIF) [file pone.0185372.s001.tif]

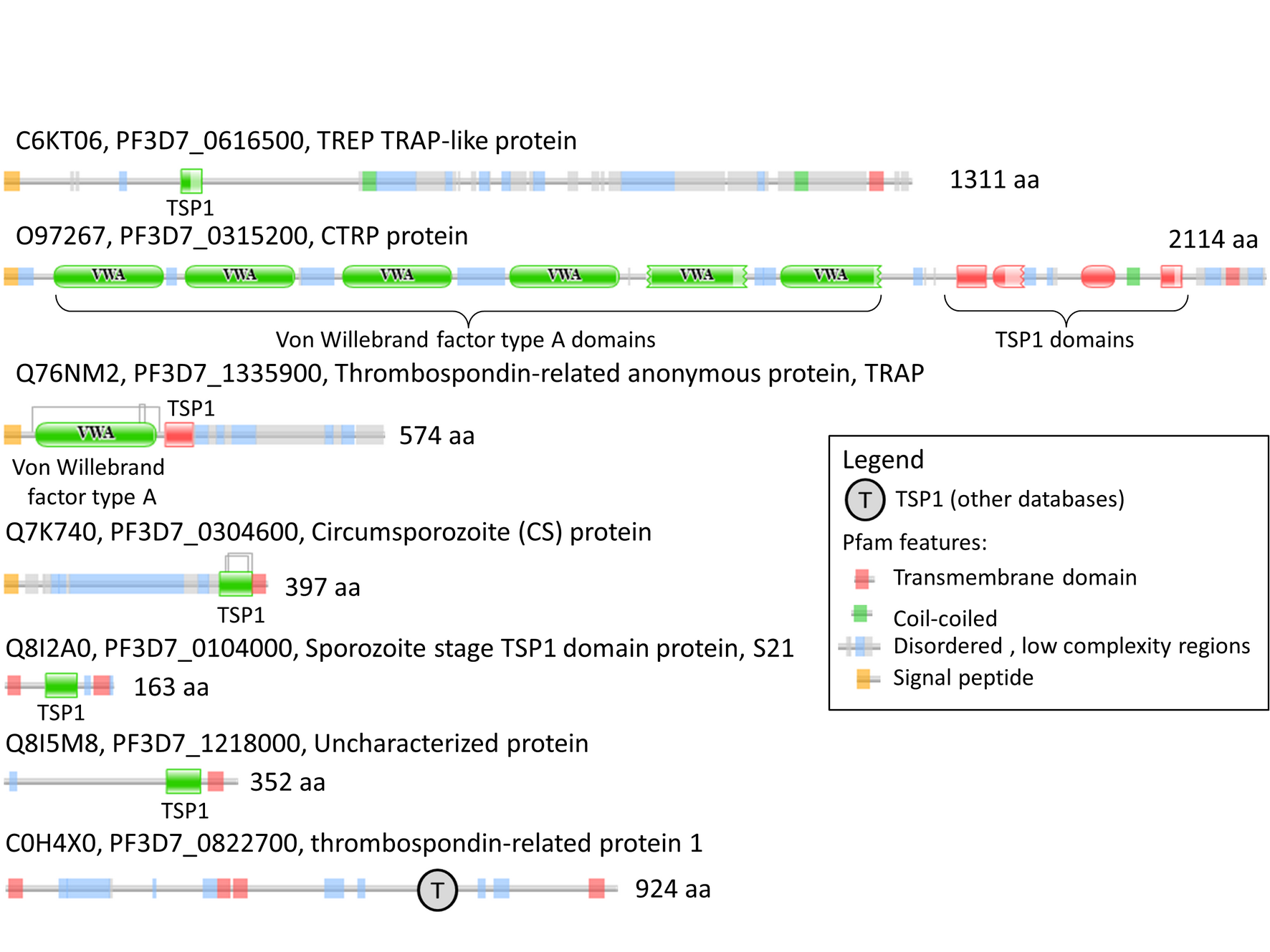

Supplement: S2 Fig — Structural description was recovered at Pfam database. Additional predictions were recovered from InterPro database. (TIF) [file pone.0185372.s002.tif]

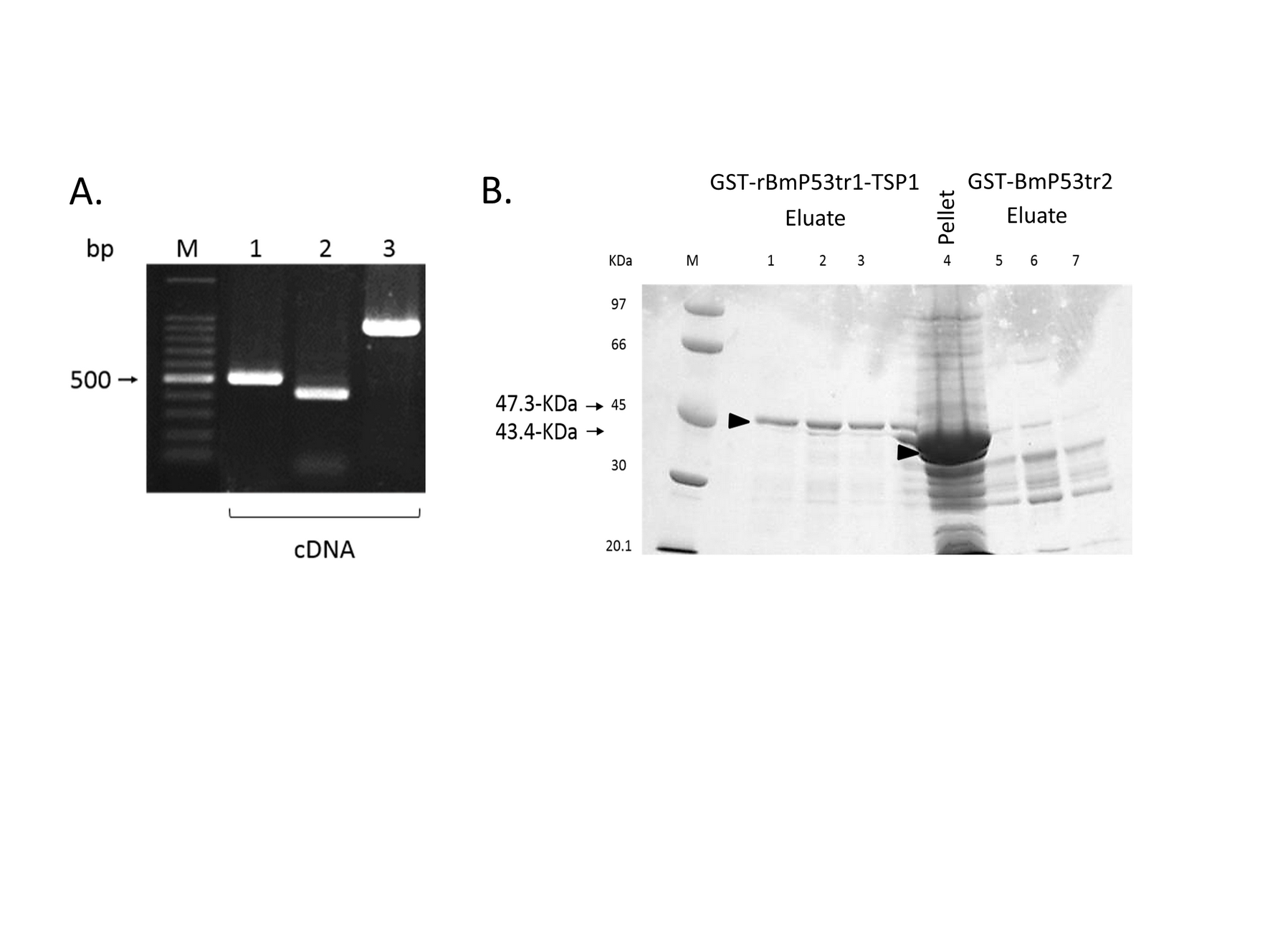

Supplement: S3 Fig — (A) Transcriptional analysis of B. microti P53 gene truncates by RT-PCR. Lane M: DNA ladder, lane 1: BmP53tr1-TSP1 mRNA, 498 bp, lane 2: BmP53tr2 mRNA, 408 bp and lane 3: BmSA1, 909 bp. (B) SDS-PAGE of expressed GST-fused recombinant BmP53 truncates stained by Coomassie blue stain. Lane M: Low molecular weight marker. Lanes 1, 2 and 3: Different elutes contain purified soluble expressed GST-rBmP53tr1-TSP1 from lysed bacteria supernatant, 47.3-KDa. Lane 4: Insoluble expressed GST-rBmP53tr2 protein in E. coli pellet, 43.4-KDa. Lanes 5, 6 and 7: Different elutes missing the non-purified insoluble expressed GST-rBmP53tr2. (TIF) [file pone.0185372.s003.tif]

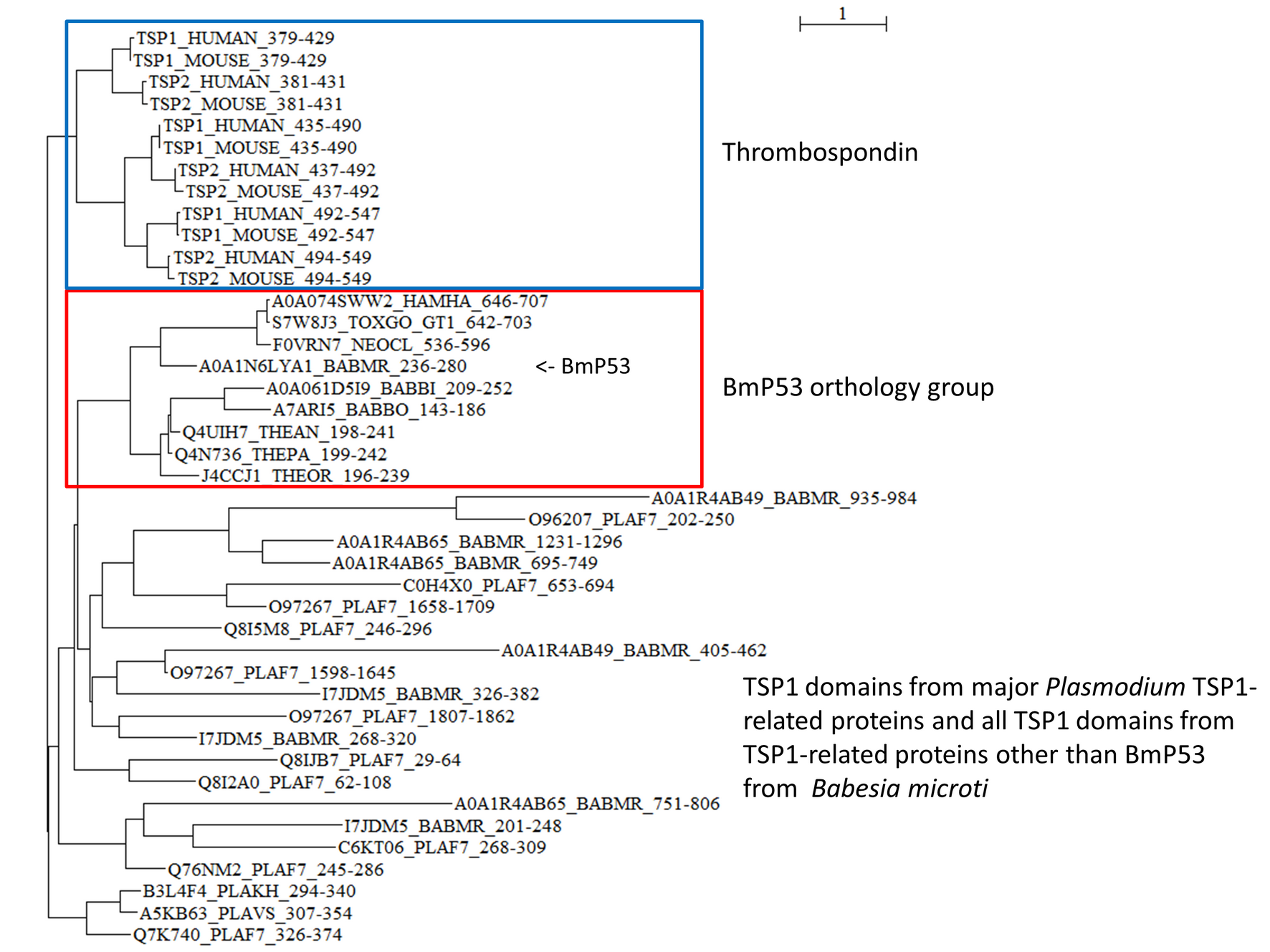

Supplement: S4 Fig — TSP1 domains are identified by their coordinates and proteins by their UNIPROT ID and UNIPROT species ID: BABBI, Babesia bigemina; BABBO: Babesia bovis; BABMR, Babesia microti; HAMHA, Hammondia hammondi; NEOCL, Neospora caninum; PLAF7, Plasmodium falciparum (isolate 3D7); PLAKH, Plasmodium knowlesi (strain H); PLAVS, Plasmodium vivax (strain Salvador I); THEAN, Theileria annulata; THEOR, Theileria orientalis (strain Shintoku); THEPA, Theileria parva; TOXGO, Toxoplasma gondii (isolate GT1). The scale top right-hand corner of the tree indicates the number of substitutions between sequences. (TIF) [file pone.0185372.s004.tif]

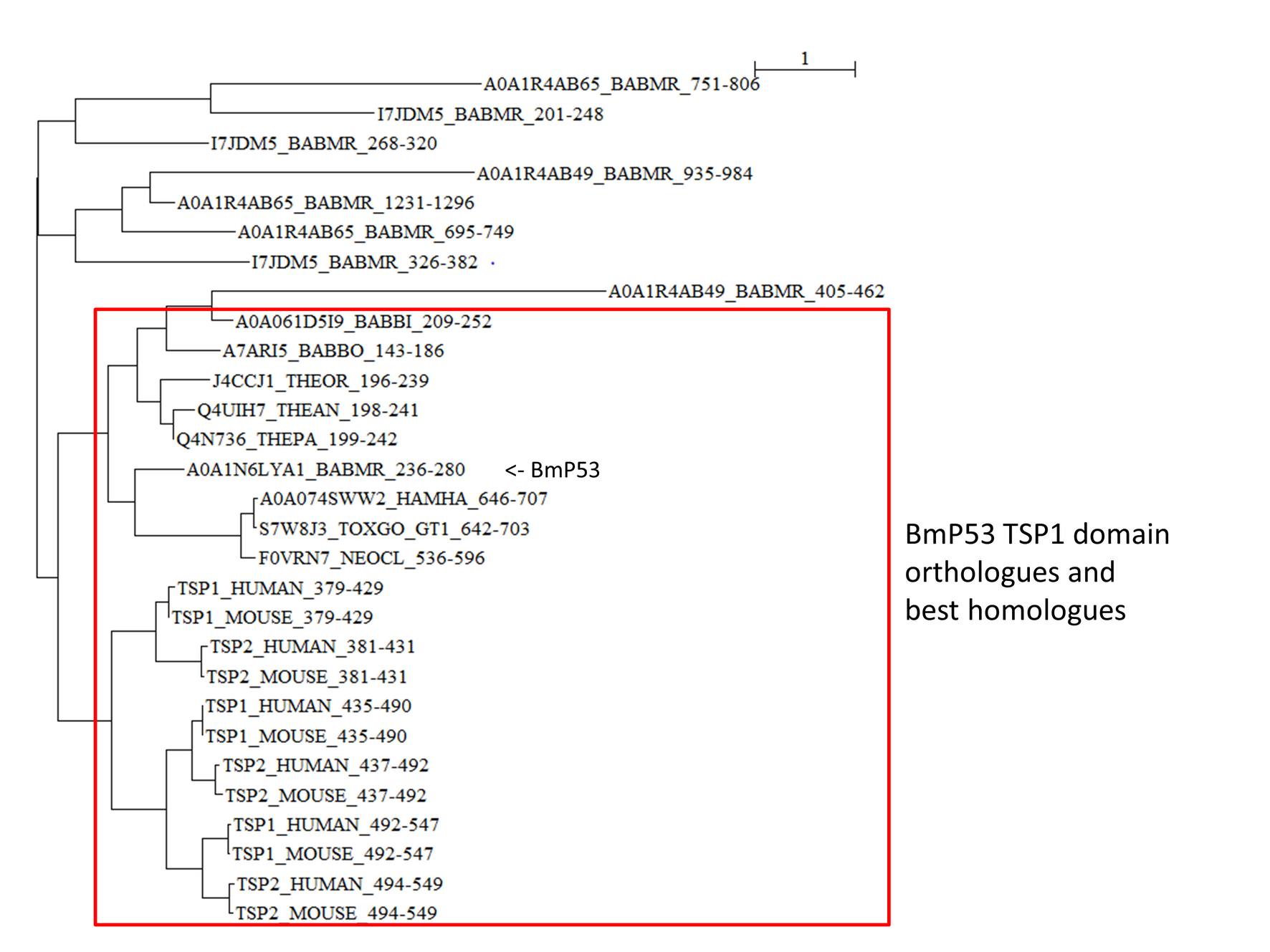

Supplement: S5 Fig — TSP1 domains are identified by their coordinates and proteins by their UNIPROT ID and UNIPROT species ID: BABBI, Babesia bigemina; BABBO: Babesia bovis; BABMR, Babesia microti; HAMHA, Hammondia hammondi; NEOCL, Neospora caninum; THEAN, Theileria annulata; THEOR, Theileria orientalis (strain Shintoku); THEPA, Theileria parva; TOXGO, Toxoplasma gondii (isolates GT1). The scale top right-hand corner of the tree indicates the number of substitutions between sequences. (TIF) [file pone.0185372.s005.tif]
